# Survival benefit from abemaciclib in non–small cell lung cancer by Kirsten rat sarcoma*–*mutation gene expression subtype: retrospective analysis from the JUNIPER Trial

**DOI:** 10.3389/fonc.2025.1461530

**Published:** 2025-03-31

**Authors:** Jiangang Liu, Hong Wang, Amit Aggarwal, Maria Jesus Ortiz-Ruiz, Elisabet Zapatero-Solana, Maria Jose Lallena, Sandra Peregrina, Gloria Martinez del Hoyo, Susana Velasco, Philip J. Ebert, Xueqian Gong, Anwar M. Hossain, Shawn T. Estrem

**Affiliations:** ^1^ Eli Lilly and Company, Indianapolis, IN, United States; ^2^ Eli Lilly and Company, Madrid, Spain; ^3^ Cell Signaling and Immunometabolism Laboratory, Centro de Investigaciones Oncológicas, Madrid, Spain

**Keywords:** biomarkers, CDK4, CDK6, *KRAS*, non-small cell lung cancer clinical trial registration: NCT02152631

## Abstract

**Purpose:**

JUNIPER, a randomized, phase III trial of patients with stage IV non–small cell lung cancer and a detectable Kristen rat sarcoma (*KRAS*) mutation in codons 12 or 13 whose condition progressed after platinum-based chemotherapy and up to 1 additional therapy (could include immune checkpoint inhibitors), reported prolonged progression-free survival (PFS) but not overall survival (OS) among patients who were receiving abemaciclib versus those who were receiving erlotinib. To establish whether certain patient subgroups received an OS benefit from the addition of abemaciclib to best supportive care, JUNIPER patients were retrospectively evaluated for *KRAS* co-mutation gene expression subtype, and abemaciclib efficacy was assessed for each patient subgroup.

**Materials and methods:**

Of the 453 patients enrolled in the JUNIPER trial, tumor specimens for biomarker analysis were available for 148 (abemaciclib arm, n=79; erlotinib arm, n=69). Samples were profiled for gene expression and classified into 3 previously identified expression subtypes (KL, KP, and K). Tumor response, OS, and PFS were assessed within each subtype.

**Results:**

Retrospective analyses of expression subtypes revealed an OS advantage for patients with KL subtype tumors who were receiving abemaciclib versus those with the KL subtype who were receiving erlotinib (median, 13.05 vs 5.65 months; hazard ratio, 0.25; 95% confidence interval, 0.09–0.73; *P*=.011). KL and KP expression subtype groups derived a PFS benefit from abemaciclib versus erlotinib (KL median, 6.64 vs 2.1 months; hazard ratio, 0.12; 95% confidence interval, 0.03–0.41; *P*=.001, and KP median, 5.52 vs 2.24 months; hazard ratio, 0.44; 95% confidence interval, 0.23–0.84; *P*=.013). Patients with K subtype tumors received no OS or PFS benefit from abemaciclib treatment.

**Conclusions:**

Patients with KL expression subtype tumors may derive better OS and PFS from abemaciclib versus erlotinib in *KRAS*-mutated non–small cell lung cancer. These results should be further validated in an independent dataset.

## 1 Introduction

Non–small cell lung cancer (NSCLC) is a heterogeneous disease with numerous histologic and molecular subtypes (1). These molecular subtypes have distinct genomic alterations and clinical outcomes, which can be associated with differential response to treatment (2). Kirsten rat sarcoma (*KRAS*), the most commonly mutated oncogene in NSCLC, has a prevalence of 29% in lung adenocarcinoma (3). Recently, 3 different *KRAS* subtypes—KL, KP, and K—have been identified in lung adenocarcinoma based on gene expression and transcriptional profiling. Each is associated with co-mutations (4, 5); *STK11*/*LKB1* and *TP53* are co-mutations in KL and KP, respectively, whereas K includes *CDKN2A/B* inactivation and low expression of *NKX2-1* (TTF1) transcription factor (4, 5). This classification distinguishes tumor subsets with distinct biology, immune profiles, and therapeutic vulnerabilities (6).

Recent reports detail co-mutation-dependent treatment variability in *KRAS*-mutant NSCLC. A retrospective analysis demonstrated that immune checkpoint inhibitors provide variable benefit for *KRAS*-mutant NSCLC, with the *KRAS* plus *STK11*/*LKB1* co-mutation (KL subtype) being resistant to this treatment class (7). Additionally, the 2 recently approved therapies targeting *KRAS* G12C, the most common *KRAS* genetic variant detected in NSCLC, may provide variable benefit based on the specific co-mutations (6, 8).

The synthetic lethal interaction between *KRAS* mutations and cyclin-dependent kinase (CDK) 4 and 6 inhibition highlights a promising therapeutic strategy for using CDK4/6 inhibitors in NSCLC (9). The CDK4/6-retinoblastoma pathway is frequently dysregulated in NSCLC, further emphasizing its potential as an attractive therapeutic target (9, 10). Abemaciclib, a CDK4/6 inhibitor, has been approved by the United States Food and Drug Administration as monotherapy and in combination with endocrine therapy for women with hormone receptor–positive, human epidermal growth factor receptor-2–negative metastatic breast cancer across various treatment lines and settings and in patients with high-risk hormone receptor–positive, human epidermal growth factor receptor-2–negative early breast cancer (11–16).

In the phase III JUNIPER trial, abemaciclib did not demonstrate an overall survival (OS) benefit in patients with NSCLC who harbored a *KRAS* mutation (17). However, improved progression-free survival (PFS) and objective response rate (ORR) in the abemaciclib arm compared with that in the erlotinib arm suggested some activity. The objective of this retrospective analysis was to evaluate the clinical outcome of participants from the JUNIPER trial in the context of mRNA expression–based subtypes to identify potentially predictive associations.

## 2 Methods

### 2.1 Patients and study design

The JUNIPER study design (NCT02152631) has been previously published (18). Briefly, JUNIPER was a randomized, open-label, parallel, comparator-controlled phase III trial that evaluated the efficacy and safety of abemaciclib versus erlotinib in patients with stage IV NSCLC with a detectable *KRAS* mutation (at the time of study design, erlotinib was used in second-line/subsequent-line treatment in NSCLC without any limitation regarding epidermal growth factor receptor (*EGFR*) mutation status) (17). Patients were stratified by number of prior chemotherapy regimens (1 vs 2), Eastern Cooperative Oncology Group performance status (ECOG PS [0 vs 1]), sex, and *KRAS* mutation (G12C vs all others) and were randomized 3:2 to receive best supportive care plus either abemaciclib (200 mg twice daily) or erlotinib (150 mg twice daily) for a 28-day cycle. Inclusion criteria were age ≥18 years; a confirmed diagnosis of stage IV NSCLC; a *KRAS* codon 12 or 13 mutation; and disease progression following platinum-based chemotherapy with the receipt of at least 1 other systemic therapy or deemed ineligible for further chemotherapy. The protocol was approved by each participating institutional ethical review board and was conducted in accordance with the ethical principles of the Declaration of Helsinki, Council for International Organizations of Medical Sciences International Ethical Guidelines, and good clinical practice. All patients provided written informed consent prior to treatment.

### 2.2 *KRAS* mutation analysis

At screening, *KRAS* mutation status was determined by the central laboratory using the QIAGEN^®^ therascreen^®^ *KRAS* Rotor-Gene Q Polymerase Chain Reaction (Qiagen, Germany) kit for NSCLC with formalin-fixed paraffin-embedded (FFPE) tumor tissue collected by surgical biopsy, fine needle aspiration, or core needle biopsy. FFPE tumor tissue samples were obtained prior to enrollment from patients who consented to participate.

### 2.3 RNA sequence

Archival FFPE tumor tissue samples from 167 patients were macro-dissected to minimize the amount of adjacent normal tissue (Almac Diagnostics Ltd, USA). RNA extraction was performed using the Qiagen RNeasy extraction kit (Qiagen, Germany) followed by quality assessments using NanoDrop (Thermo Fischer Scientific, USA), Bioanalyzer (Agilent, USA), and RNA sequencing (an Almac Diagnostics Ltd, USA proprietary quality control step). Illumina TruSeq RNA Exome sequencing was performed by Almac with the Illumina TruSeq RNA Sample Preparation Kit v2 as described previously (4). Paired-end sequencing with a read length of 100 base pairs and targeted read depth of 50 million reads/sample was performed. Data were filtered to remove genes with fewer than 5 counts across 80% of the samples from the analysis. The resulting data were quantile-normalized and summarized across samples. Analyses were performed using the programming language R (version 3.1; http://www.r-project.org). Quality control failures were flagged for 11 tumor samples due to low overall read depth, indicating insufficient input material or other sources of assay failures. Raw counts for 156 samples were log2 transformed and normalized. Eight outlier samples were identified using principal component analysis of normalized counts and were removed from further analysis, leaving 148 translational marker-evaluable samples.

### 2.4 Gene expression subtype classification

We applied a single sample predictor (SSP) algorithm to obtain subtype labels for the 148 translational marker-evaluable samples according to the Skoulidis classification (5). The SSP utilizes correlations between each sample and the molecular subtype “centroids,” which are representative vectors of average gene expression values for subtype-specific genes (19). To classify a new sample, its distance to each of the centroids is calculated and it is assigned to the subtype corresponding to the nearest centroid (20). For the Skoulidis expression subtypes (KL, KP, and K), the centroids consisting of an average expression of 384 genes generated from RNA sequences were used (4). Pearson correlation coefficient was used for the samples of each subtype centroid to assign the sample to 1 of 3 tumor subtypes (KL, KP, and K). We also calculated the difference between the highest and second-highest correlations to the centroids for each sample. Additionally, the highest correlations to these centroids were computed for virtual tumor sample arrays obtained by random permutations of the data for each gene. Tumors with a correlation ≥0.15 (a default parameter from the “CMSclassifier” R package; https://github.com/Sage-Bionetworks/CMSclassifier/blob/master/R/cmsClassifier.R) with any of the 3 centroids were classified, and tumors with a correlation of <0.15 remained unclassified. Each sample was assigned to the subtype with which it had the highest Pearson correlation coefficient.

To classify the tumors that remained unclassified by SSP, random forest, a well-established machine-learning method that operates by generating multiple bootstrapped versions of the training data to fit a decision tree to each of these bootstraps, was implemented (21). The random forest algorithm has been well studied in the context of gene expression classifiers, as it performs well with highly correlated, high-dimensional data and is less prone to overfitting because of the averaging effect across many models (22, 23). RNA sequence data of 67 TCGA samples with KL, KP, or K subtype labels were split at the ratio of 3:1 for training and validation, and a random forest classifier was generated from 500 balanced bootstraps of the training data (10). After model training, the classifier was applied to the validation samples and performance metrics were computed (sensitivity, specificity, and balanced accuracy). When applied to the validation data, the classifier demonstrated robust performance and evidenced subtype-specific characteristics.

The RNAseq data (reads per kilobase million) of 43 *KRAS*-mutated NSCLC cell lines were downloaded from Cancer Cell Line Encyclopedia (24). Gene expression profiles from the cell lines were correlated to the centroids reported by Skoulidis et al. for each of the KL, KP, and K subtypes using the same method used to classify the primary tumors. To assess the cell line subtype vulnerabilities, the antiproliferative sensitivity (half-maximal inhibitory concentration [IC50]) of abemaciclib for the 43 *KRAS*-mutated cell lines was retrieved from a novel cancer cell sensitivity profiling (CCSP) assay format developed internally (25).

### 2.5 Generation of STK11/LKB1-overexpressing cells

Retroviruses were produced by transfection of HEK293T cells with pBABE-FLAG-LKB1 overexpression plasmid (plasmid 8592, Addgene, USA; gift from Lewis Cantley) (26) and packaging plasmid encoding VSV-G, gag-pol, using Lipofectamine 2000 (Invitrogen, USA). Viral particle–containing supernatants were collected 48 hours post-transfection, filtered through a 0.45 μm syringe filter, and used for infection immediately.

NCI-H1944 cells were plated the day before infection at approximately 70% confluence in complete growth medium (RPMI 1640 medium, ATCC modification 30-2001) and allowed to adhere overnight. The next day, cells were infected with viral supernatants in the presence of 6 μg/mL polybrene (Sigma-Aldrich, USA). After infection, successfully transduced cells were obtained by selection with 2 µg/mL puromycin (Millipore, USA).

### 2.6 Propidium iodide cell viability

Cells were plated at 2000 cells per well in 96-well plates in a total volume of 100 μL of growth media alone (RPMI 1640, 10% fetal bovine serum, 1% penicillin/streptomycin). Twenty-four hours post-seeding, cells were treated with either dimethyl sulfoxide or decreasing concentrations of test compounds in the range of 20 μM–1 nM. Test compounds were prepared at 2 times concentration in 100 µL of growth media using a dilution factor of 1:3 with a total of 0.4% dimethyl sulfoxide (0.2% final concentration in cells). After cells were incubated at 37°C for 2 doubling times (72 hours), cells were fixed with 70% ice-cold ethanol for 30 minutes and then treated with RNAse and nuclei stained with propidium iodide in phosphate-buffered saline for 1 hour. Cell nuclei per well were counted using Acumen Explorer™ (STP LabTech Ltd, UK) to determine the number of cells remaining after treatment. For data analyses, IC50 was determined by curve fitting to a 4-parameter logistic equation for each output normalized to dimethyl sulfoxide using GraphPad Prism^®^.

### 2.7 Western blotting

Cells were washed with phosphate-buffered saline and lysed in ice-cold lysis buffer (140 mM NaCl, 10 mM ethylenediaminetetraacetic acid, 10% glycerol, 1% Nonidet P40, 20 mM Tris, pH 8.0, cOmplete™ Mini Protease and PhosSTOP phosphatase Inhibitor Cocktail). Lysates were immediately frozen at -−-80°C and centrifuged at 12,500 rpm at 4°C for 10 minutes before protein quantification. A total of 40 µg of each sample quantified by Pierce Protein Assay Reagent (620 nm) (Thermo Fisher Scientific, USA) was loaded into Precast Midi Protein Gels (Bio-Rad, 5671095). Electrophoresis was run for 1.5 hours at 120 V in Running Buffer 1X (Sigma -Aldrich, USA), and protein transference was performed using Trans-Blot Turbo Transfer System and pre-assembled transfer packs (0.2 µm nitrocellulose membranes; Bio-Rad, USA). Immunoblotting was performed in a blocking buffer of 2% bovine serum albumin/phosphate-buffered saline-Tween 20 and detected by anti-LKB1 antibody (Cell Signaling Technology, USA) using enhanced chemiluminescence-horseradish peroxidase substrate (Thermo Fisher Scientific, USA) on Amersham Imager 600. Vinculin (Sigma-Aldrich, USA) was used as a loading control. Since cells were cultured under puromycin pressure and viability assays were performed in growth media alone, protein expression analyses were done in cells cultured with and without puromycin selection.

### 2.8 Statistical analyses

Efficacy outcomes, including ORR, disease control rate (DCR), and time-to-event variables (OS and PFS) were assessed in abemaciclib- versus erlotinib-treated patients, in the translational research (TR) population (patients with evaluable gene expression data available for association analysis with clinical efficacy), and for each of the subtypes (KL, KP, and K). A logistic regression model stratified by sex, ECOG PS, number of prior chemotherapies, and *KRAS* mutation was used to investigate the relationship between ORR, DCR, and treatment for each expression subtype; *P*-values were derived from the likelihood ratio test. ORR was defined as the combined rates of complete response and partial response (PR), and DCR was defined as the combined rates of complete response, PR, and stable disease (SD). The Kaplan-Meier product limit method was used to estimate the OS and PFS survival curves; 95% confidence intervals (CIs) for median OS and median PFS were computed by the Brookmeyer and Crowley method (27). A stratified Cox proportional hazards model was used to compare the treatment effect in each expression subtype, hazard ratio (HR) estimates, and their 95% CIs. Log-rank test *P*-values were reported on the Kaplan-Meier plot.

In addition, multivariable Cox proportional hazards models were performed including treatment, expression subgroups, treatment-by-subgroup interactions, key clinical covariates (age, prior immunotherapy, smoking status), and stratification factors as explanatory variables within each expression subtype. Univariate subgroup analyses for key clinical covariates and stratification factors were performed for each expression subtype and reported on forest plots. The effect size was calculated using a linear mixed-effects model implemented with the lme4 R package (version 1.1.26). All analyses in this study were exploratory in nature, and no multiplicity adjustments were performed.

## 3 Results

### 3.1 Translational research population

**Figure 1:**
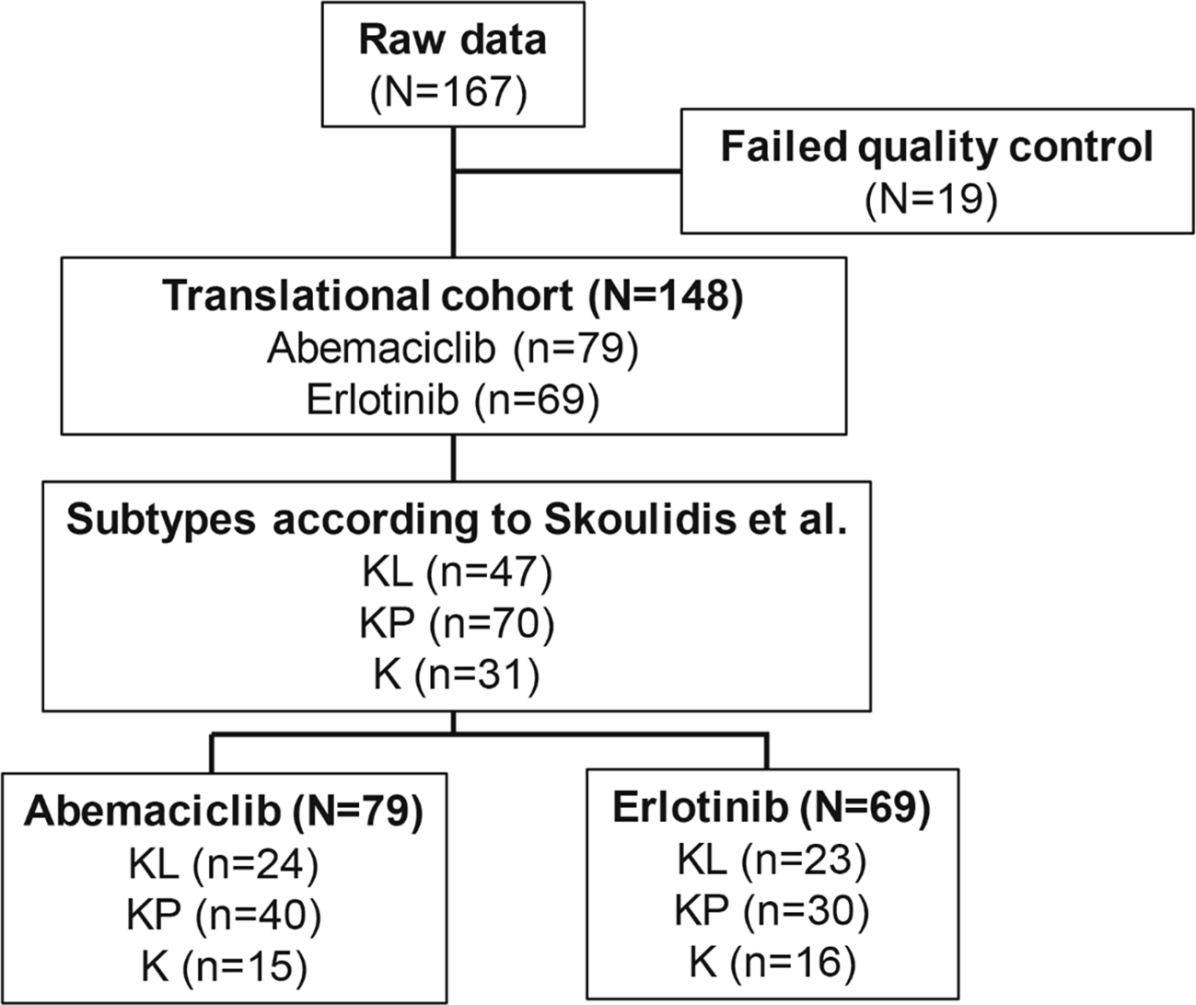
Flow diagram demonstrating sample derivation for biomarker analyses. N, number of patients in the population; n, number of patients in the specified category.

RNA was obtained from 167 archival JUNIPER FFPE tumor samples, with high-quality data (data that passed quality control) from 148 samples (TR population; abemaciclib arm, n=79; erlotinib arm, n=69). Sample flow through the study is shown in **Figure 1**. Baseline demographic characteristics in the TR and intent-to-treat (ITT) populations were similar (**Table 1**), except that the TR population underrepresented Asian patients in the ITT population (2.5% and 7.2% for TR vs 20.0% and 22.4% for ITT in the abemaciclib and erlotinib arms, respectively). Within the TR population, notable differences in percentages were observed between the 2 arms for the categories of KRAS G12C mutation and ECOG PS of 0. In the abemaciclib arm, 63.3% had the KRAS G12C mutation (median OS: 7.61 months; median PFS: 3.91 months), compared to 49.3% in the erlotinib arm (median OS: 6.05 months; median PFS: 1.92 months; χ²test, *P*= .12). For ECOG PS of 0, 26.6% were in the abemaciclib arm (median OS: 10.2 months; median PFS: 6.61 months) while 18.8% were in the erlotinib arm (median OS: 11.9 months; median PFS: 2.20 months; χ² test, *P*=.357). However, these differences were not statistically significant. For participants receiving abemaciclib, numerically longer OS (**Supplementary Figure 1A**) and PFS (**Supplementary Figure 1B**) were observed in the TR population than in the ITT population, while erlotinib OS was numerically shorter in the TR verses the ITT population, and PFS was comparable between the TR and ITT populations.

**Table 1:** Baseline demographic and disease characteristics of the TR and ITT populations.

Characteristic	TR Population		ITT Population	
	Abemaciclib (N=79)	Erlotinib (N=69)	Abemaciclib (N=270)	Erlotinib (N=183)
Sex, male, n (%)	45 (57.0)	39 (56.5)	163 (60.4)	109 (59.6)
Age, years, median (range)	61 (36–77)	64 (36–79)	62 (36–89)	63 (39–83)
Age category, ≥65 years, n (%)	29 (36.7)	32 (46.4)	108 (40.0)	77 (42.1)
Region, n (%)				
Asia	2 (2.5)	5 (7.2)	54 (20.0)	41 (22.4)
Europe	60 (75.9)	51 (73.9)	160 (59.3)	106 (57.9)
North America	15 (19.0)	11 (15.9)	48 (17.8)	29 (15.8)
Other	2 (2.5)	2 (2.9)	8 (3.0)	7 (3.8)
Pathological diagnosis, n (%)				
Adenocarcinoma	71 (89.9)	60 (87.0)	243 (90.0)	165 (90.2)
Squamous	5 (6.3)	5 (7.2)	9 (3.3)	6 (3.3)
Other	3 (3.8)	4 (5.8)	18 (6.7)	12 (6.5)
Smoking status, n (%)				
Past smoker	58 (73.4)	51 (73.9)	198 (73.3)	127 (69.4)
Current smoker	13 (16.5)	11 (15.9)	44 (16.3)	28 (15.3)
Never smoked	8 (10.1)	6 (8.7)	28 (10.4)	26 (14.2)
Missing	0	1 (1.4)	0	2 (1.1)
ECOG PS, n (%)				
0	21 (26.6)	13 (18.8)	64 (23.7)	44 (24.0)
1	58 (73.4)	56 (81.2)	206 (76.3)	139 (76.0)
*KRAS*-mutant (BL), n (%)				
G12C	50 (63.3)	34 (49.3)	145 (53.7)	96 (52.5)
Others	29 (36.7)	35 (50.7)	125 (46.3)	87 (47.5)
Prior chemotherapy				
One	21 (26.6)	19 (27.5)	59 (21.9)	43 (23.5)
Two	58 (73.4)	50 (72.5)	211 (78.1)	139 (76.0)
Missing	0	0	0	1 (0.5)
Prior immunotherapy				
Yes	11 (13.9)	8 (11.6)	46 (17.0)	30 (16.4)
No	68 (86.1)	61 (88.4)	224 (83.0)	153 (83.6)

BL, baseline; ECOG PS, Eastern Cooperative Oncology Group performance status; G12C, mutation in codon 12 of the *KRAS* gene resulting in an amino acid substitution from glycine to cysteine; ITT, intent-to-treat; *KRAS*, Kirsten rat sarcoma; N, number of patients in the population; n, number of patients in the specified category; TR, translational research.

### 3.2 Tumor subtype classification

**Figure 2:**
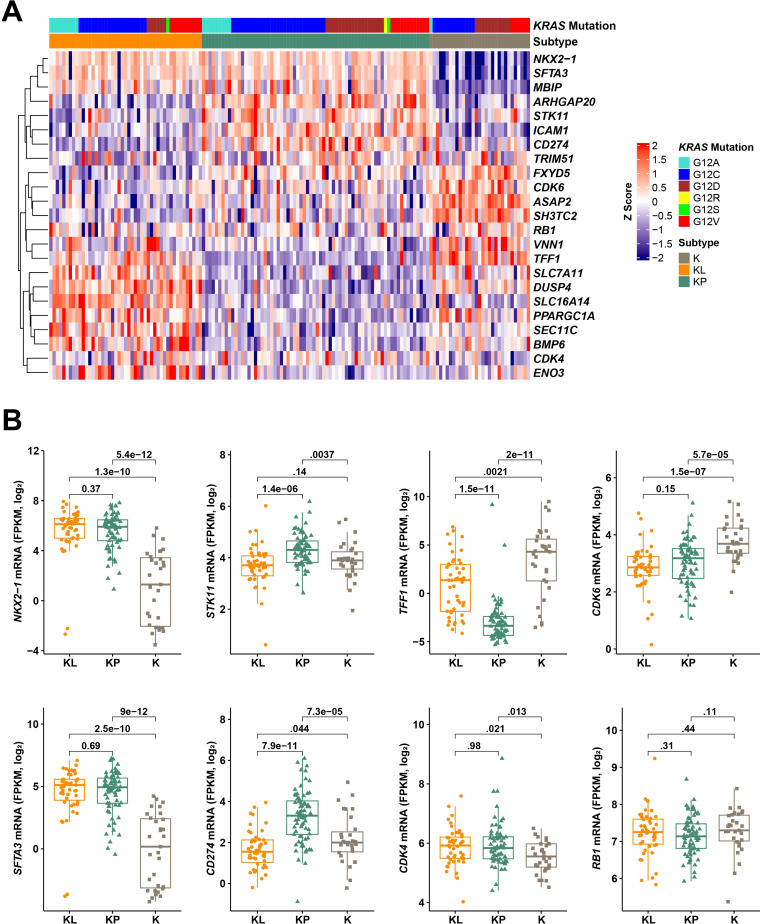
*KRAS*-mutant expression subtypes assigned based on Skoulidis et al.’s method. Heat map shows gene expression (z scores) of 18 core classifier genes and 3 additional characteristic genes (*NKX2-1*, *STK11*, *CD274*) identified by Skoulidis et al. and 3 abemaciclib target-related genes. Genes (rows) were clustered using Pearson correlation distance and hierarchical clustering. Samples (columns) were grouped by subtypes assigned based on Skoulidis et al.’s method **(A)**. mRNA expression of 5 subtype specific genes (*NKX2-1*, *SFTA3*, *TFF1*, *CD274* [PD-L1], *STK11*) and 3 abemaciclib target-related genes (*CDK4*, *CDK6*, *RB1*) in *KRAS*-mutant lung adenocarcinoma subsets. The Wilcoxon test was used for statistical comparison between the 3 subgroups **(B)**. CDK, cyclin-dependent kinase; FPKM, fragments per kilobase of transcript per million mapped reads; G12C, mutation in codon 12 of the *KRAS* gene resulting in an amino acid substitution from glycine to cysteine; ITT, intent-to-treat; *KRAS*, Kirsten rat sarcoma; mRNA, messenger ribonucleic acid; PD-L1, programmed death-ligand 1; RB, retinoblastoma; RPM, revolutions per minute; *SFTA3*, surfactant associated 3; *STK11*, serine/threonine kinase 11; TFF1, trefoil factor 1.

Of the 148 samples, 113 (76.4%) were assigned to an expression subtype; 35 remained unclassified by SSP. Random forest was then applied to classify the remaining samples. The final classification assigned 70 KP (46.5%), 47 KL (32.7%), and 31 K (20.8%) subtypes. Hierarchical clustering with 18 core classifier genes, 3 additional characteristic genes (*NKX2-1, STK11, CD274*) identified by Skoulidis et al. (5), and 3 abemaciclib target-related genes (CDK4, CDK6, RB1) displayed as a heatmap (colored with a gradient from blue for low expression to red for high expression) highlights the selected subtype-specific gene enrichment in multiple subtypes (**Figure 2A**).

In the K subtype, the *NKX2*-1 (*TTF1*) transcription factor showed low or no expression in log_2_-transformed fragments per kilobase of transcript per million mapped reads [FPKM]: 1.08 vs 5.61 for KL, 5.54 for KP; *P*<1.3e–10), along with its downstream lung-specific gene *SFTA3* (log_2_-transformed FPKM: −0.09 vs 4.52 for KL, 4.55 for KP; *P*<2.5e–10). The immune checkpoint ligand *CD274* (programmed death-ligand 1) was expressed at a higher level in KP tumors (log_2_-transformed FPKM: 2.16 for K, 1.66 for KL, 3.27 for KP; P=7.9e–11 KP vs KL). *STK11*/*LKB1* mRNA expression was lower in the KL subtype than in the KP subtype (log_2_-transformed FPKM: 3.89 for K, 3.69 for KL, 4.3 for KP; *P*=1.4e–06). *CDK4* and *CDK6* expression levels did not show statistically significant differences between the KL and KP tumors; however, patients with K tumors had lower *CDK4* (log_2_-transformed FPKM: 5.55 for K, 5.88 for KL, 5.94 for KP) and higher *CDK6* expression (log_2_-transformed FPKM: 3.75 for K, 2.86 for KL, 3.02 for KP) compared to those with other tumor subtypes (**Figure 2B**). Additionally, RB1 expression levels were relatively consistent across subtypes (log_2_-transformed FPKM: 7.27 for K, 7.23 for KL, 7.15 for KP), and *TFF1* expression was significantly lower in the KP subtype (log_2_-transformed FPKM: 3.32 for K, 0.81 for KL, −2.99 for KP; *P*=1.5e–11 KP vs KL).

### 3.3 Expression subtype and tumor response

Among abemaciclib recipients with the KL subtype, 2 (8.3%) experienced a best overall response (BOR) of PR, 20 (83.3%) had a BOR of SD, and 1 (4.2%) experienced a BOR of progressive disease (PD; **Table 2**). Among erlotinib recipients with the KL subtype, none experienced a BOR of PR, 8 (34.8%) had a BOR of SD, and 10 (43.5%) patients experienced a BOR of PD (**Table 2**). A significant treatment-arm DCR difference was observed among patients with the KL subtype (abemaciclib arm, 91.7% vs erlotinib arm, 34.8%, *P*=.001). The treatment-arm ORR difference was not statistically significant (8.3% vs 0.0%, *P*=.62; **Table 2**).

**Table 2:** Tumor subtype response to treatment.

	TR Population (N=148)		Subtype KL (N=47)		Subtype KP (N=70)		Subtype K (N=31)	
	Abemaciclib (n=79)	Erlotinib (n=69)	Abemaciclib (n=24)	Erlotinib (n=23)	Abemaciclib (n=40)	Erlotinib (n=30)	Abemaciclib (n=15)	Erlotinib (n=16)
ORR, n (%)	9 (11.4)	3 (4.3)	2 (8.3)	0 (0)	7 (17.5)	1 (3.3)	0 (0)	2 (12.5)
*P*-value	.321		.62		.7		.41	
DCR, n (%)	52 (65.8)	24 (34.8)	22 (91.7)	8 (34.8)	27 (67.5)	12 (40.0)	3 (20.0)	4 (25.0)
*P*-value	.066		.001		.291		.714	
PR, n (%)	9 (11.4)	3 (4.3)	2 (8.3)	0 (0)	7 (17.5)	1 (3.3)	0 (0)	2 (12.5)
SD, n (%)	43 (54.4)	21 (30.4)	20 (83.3)	8 (34.8)	20 (50.0)	11 (36.7)	3 (20.0)	2 (12.5)
PD, n (%)	18 (22.8)	31 (44.9)	1 (4.2)	10 (43.5)	8 (20.0)	13 (43.3)	9 (60.0)	8 (50.0)
NE, n (%)	9 (11.4)	14 (20.3)	1 (4.2)	5 (21.7)	5 (12.5)	5 (16.7)	3 (20.0)	4 (25.0)

*P* values were calculated from the χ^2^ test for categorical variables.

DCR, disease control rate; N, number of patients in the population; n, number of patients in the category; NE, not evaluable; ORR, objective response rate; PD, progressive disease; PR, partial response; SD, stable disease; TR, translational research.

Abemaciclib-treated patients with the KP subtype had numerically better DCR and ORR than erlotinib-treated patients, whereas the ORR and DCR were lower, though not statistically significant, for abemaciclib- compared to erlotinib-treated patients with the K subtype (**Table 2**). Furthermore, in the TR population, no statistically significant ORR or DCR differences between treatment arms were observed (**Table 2**).

### 3.4 Expression subtype and survival

**Figure 3:**
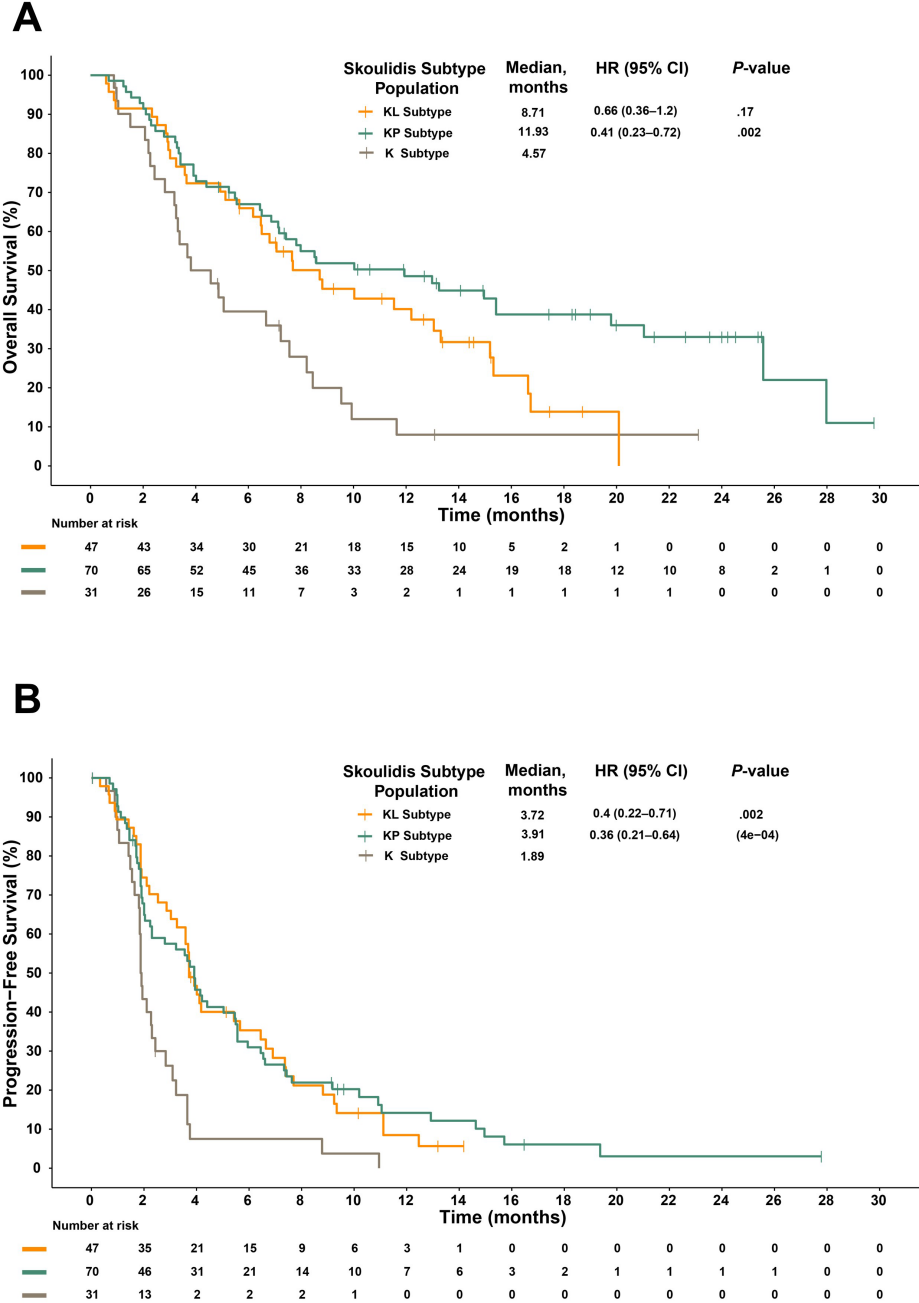
Kaplan-Meier plots of OS **(A)** and PFS **(B)** stratified by subtypes identified by Skoulidis et al. regardless of treatment arms. CI, confidence interval; HR, hazard ratio; OS, overall survival; PFS, progression-free survival.

Comparison of survival differences among the expression subtypes regardless of treatments suggests a subtype-specific prognosis. The KL, KP, and K subtypes had a median OS of 8.71, 11.93, and 4.57 months, respectively (**Figure 3A**), and a median PFS of 3.72, 3.91, and 1.89 months, respectively (**Figure 3B**). These data demonstrate that the K subtype may be a poor prognostic indicator compared to the KP subtype, which had the best OS and PFS, and the KL subtype.

**Figure 4:**
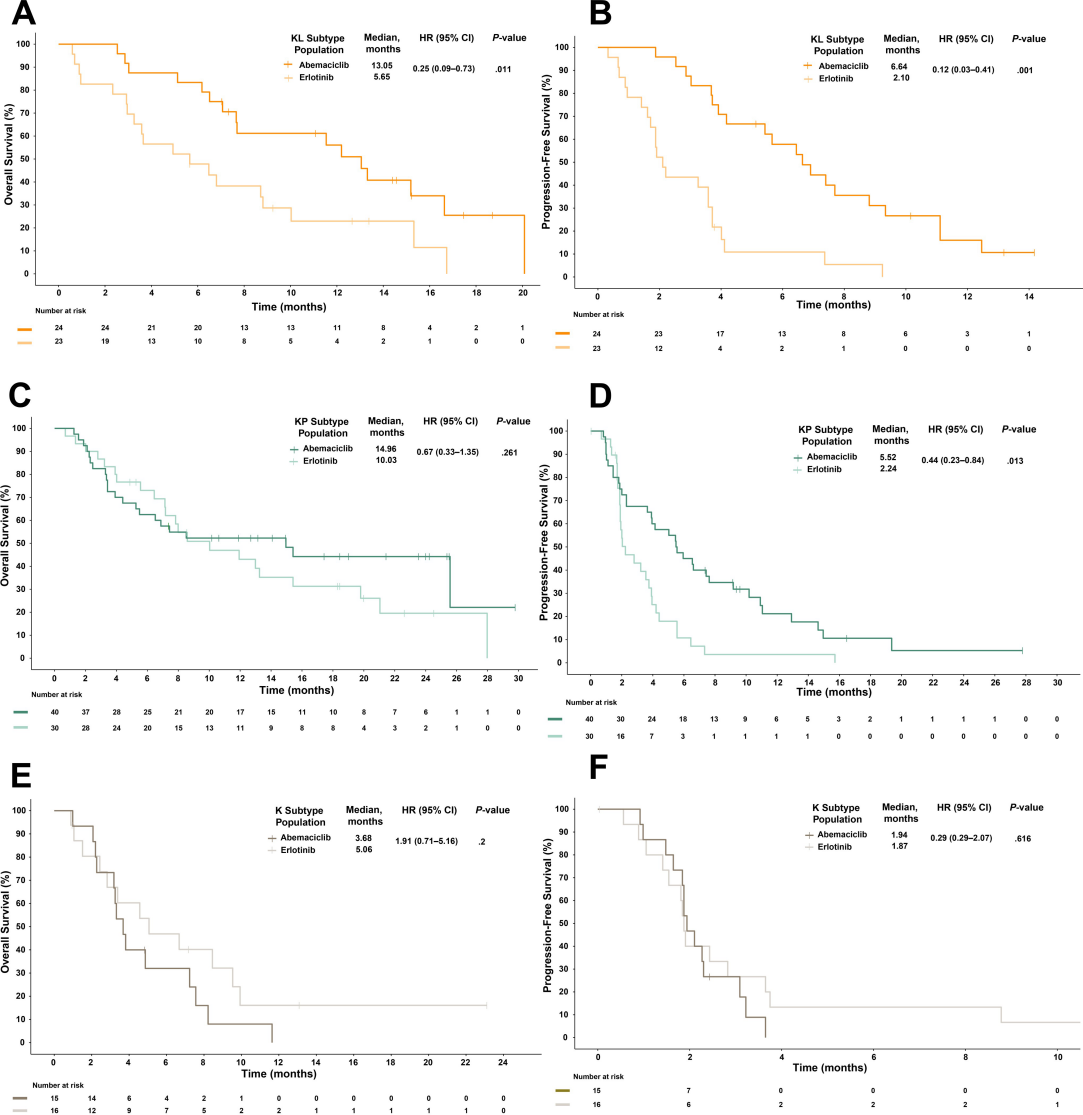
Overall survival **(A, C, E)** and progression-free survival **(B, D, F)** stratified by subtypes identified by Skoulidis et al. and treatment arm. The KL subtype is associated with better overall survival **(A)** and progression-free survival **(B)** after abemaciclib treatment than after erlotinib treatment among patients with *KRAS*-mutant lung adenocarcinoma in JUNIPER. Multivariate analysis adjusted for stratification factors was performed to compute the HRs. CI, confidence interval; HR, hazard ratio; *KRAS*, Kirsten rat sarcoma.

Patients with the KL subtype benefitted most from abemaciclib treatment (OS, 13.05 vs 5.65 months for erlotinib; HR, 0.25; 95% CI, 0.09–0.73; *P*=.011; **Figure 4A**); this differed from the KP or K subtypes, where no OS benefit was observed (**Figures 4C, E**).

Patients with the KL and KP subtypes in the abemaciclib arm had increased PFS versus those in the erlotinib arm (**Figures 4B, D**; KL: 6.64 vs 2.1 months; HR, 0.12; 95% CI, 0.03–0.49; *P*=.001; KP: 5.52 vs 2.24 months; HR, 0.44; 95% CI, 0.23–0.84; *P*=.013), while those with the K subtype had little PFS benefit (1.94 vs 1.87 months; HR, 0.78; 95% CI, 0.29–2.07; *P*=.616; **Figure 4F**).

### 3.5 Abemaciclib demonstrates selective survival benefits in the KL molecular subtype of NSCLC: a multi-model analysis

Both univariate and multivariate analyses demonstrated consistent survival benefits with abemaciclib treatment specifically in the KL molecular subtype. The multivariate Cox regression analysis confirmed the OS benefit in KL subtype patients (HR, 0.35; 95% CI, 0.15–0.79; P=.02) but not in KP (HR, 0.76; 95% CI, 0.38–1.51; P=.84) or K subtypes (HR, 1.27; 95% CI, 0.52–3.18; P=.31; **Table 3**). This pattern was also observed for PFS in the KL subtype (HR, 0.39; 95% CI, 0.20–0.72; P=.004; **Supplementary Table 1**).

**Table 3:** Multivariable analysis for OS by expression subtype.

	Subtype KL (N=47)		Subtype KP (N=70)		Subtype K (N=31)	
	Abemaciclib (n=24)	Erlotinib (n=23)	Abemaciclib (n=40)	Erlotinib (n=30)	Abemaciclib (n=15)	Erlotinib (n=16)
Patients censored, n (%)	8 (33.33)	4 (17.39)	18 (45.00)	8 (26.66)	1 (6.66)	4 (25.00)
Patients with events, n (%)	16 (66.66)	19 (82.60)	22 (55.00)	22 (73.33)	14 (93.33)	12 (75.00)
Median OS, months	13.05	5.65	14.96	10.03	3.68	5.06
HR within expression level (95% CI)	0.35 (0.15–0.79)		0.76 (0.38–1.51)		1.27 (0.52–3.18)	
*P*-value	.018		.841		.312	

Sex, ECOG PS, number of prior chemotherapies, and *KRAS* mutation were used as stratification factors in the model; age, prior immunotherapy, and smoking status were used as covariates in the model.

CI, confidence interval; ECOG PS, Eastern Cooperative Oncology Group performance status; HR, hazard ratio; *KRAS*, Kirsten rat sarcoma; OS, overall survival; N, number of patients in the population; n, number of subjects in the category.

**Figure 5:**
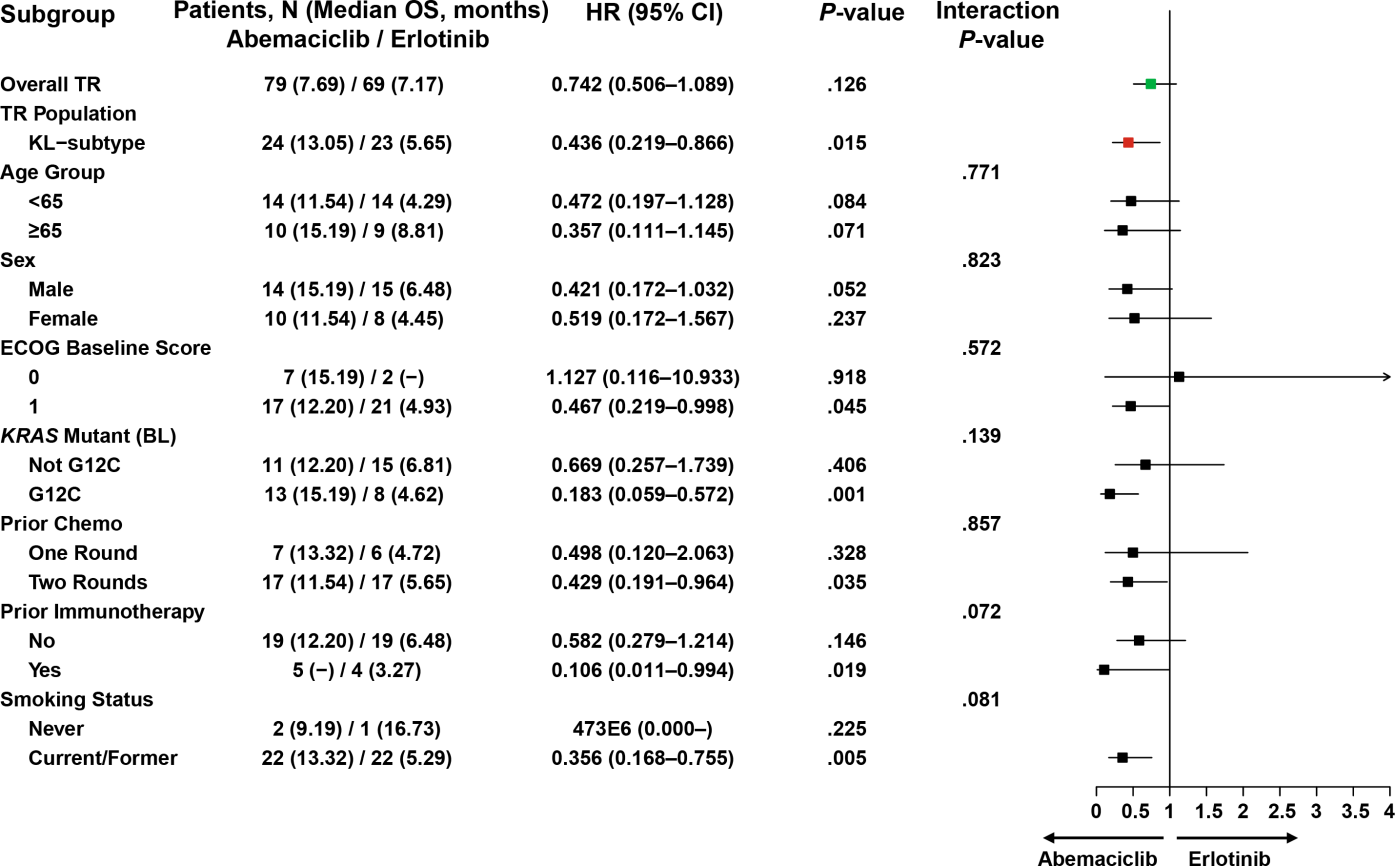
Forest plot for OS treatment effect and univariate subgroup analysis for clinical covariates and stratification factors within the KL subtype. –, Not evaluable due to the small sample size; Green: HR for the ITT population; Red: HR for the TR population; Black; subgroups; arrow line, CI out of the x-axis. Univariate analysis was performed to compute the HRs. BL, baseline; CI, confidence interval; ECOG, Eastern Cooperative Oncology Group; G12C, mutation in codon 12 of the KRAS gene resulting in an amino acid substitution from glycine to cysteine; HR, hazard ratio; ITT, intent-to-treat; *KRAS*, Kirsten rat sarcoma; OS, overall survival; TR, translational research.

The univariate subgroup analysis for clinical covariates and stratification factors in the KL subtype population showed consistency across subgroups, with expected variations. Notably, KL subtype patients with KRAS G12C mutation demonstrated superior survival with abemaciclib compared to erlotinib (median OS: 15.19 vs 4.62 months; HR, 0.183; 95% CI, 0.059–0.572; *P*=.001; **Figure 5**), despite the limited sample size (abemaciclib n=13 vs erlotinib n=8). This KRAS G12C-associated survival benefit was not observed in either the KP or K subtypes (**Supplementary Figures 2A, B**).

Effect Size Analysis: For OS, the abemaciclib arm showed a consistent positive effect (1.27–1.30) across various models (**Supplementary Figures 3A, C**), with clinical parameters like baseline ECOG PS of 1 (−3.77, *P*<.05) and stage IV disease (−6.28) demonstrating substantial negative effects, while the age category of ≥65 years showing a measurable positive effect (1.85). For PFS, the abemaciclib arm exhibited a stronger positive effect (2.95, *P*< 0.001) compared to OS (**Supplementary Figures 3B, D**), with sex (male) demonstrating a significant positive effect (1.61–1.63, *P*<.05) and Stage IV disease maintaining a consistent negative effect (−2.65 to −4.36). Notably, both KP and KL subtypes demonstrated significant effects for OS (KP: 5.81, *P*<.001; KL: 3.57, *P*<.05) and PFS (KP: 2.76, *P*<.01; KL: 2.38, *P*<.05).

### 3.6 *KRAS*-mutated NSCLC cell lines with KL expression subtypes are more sensitive to abemaciclib

**Figure 6:**
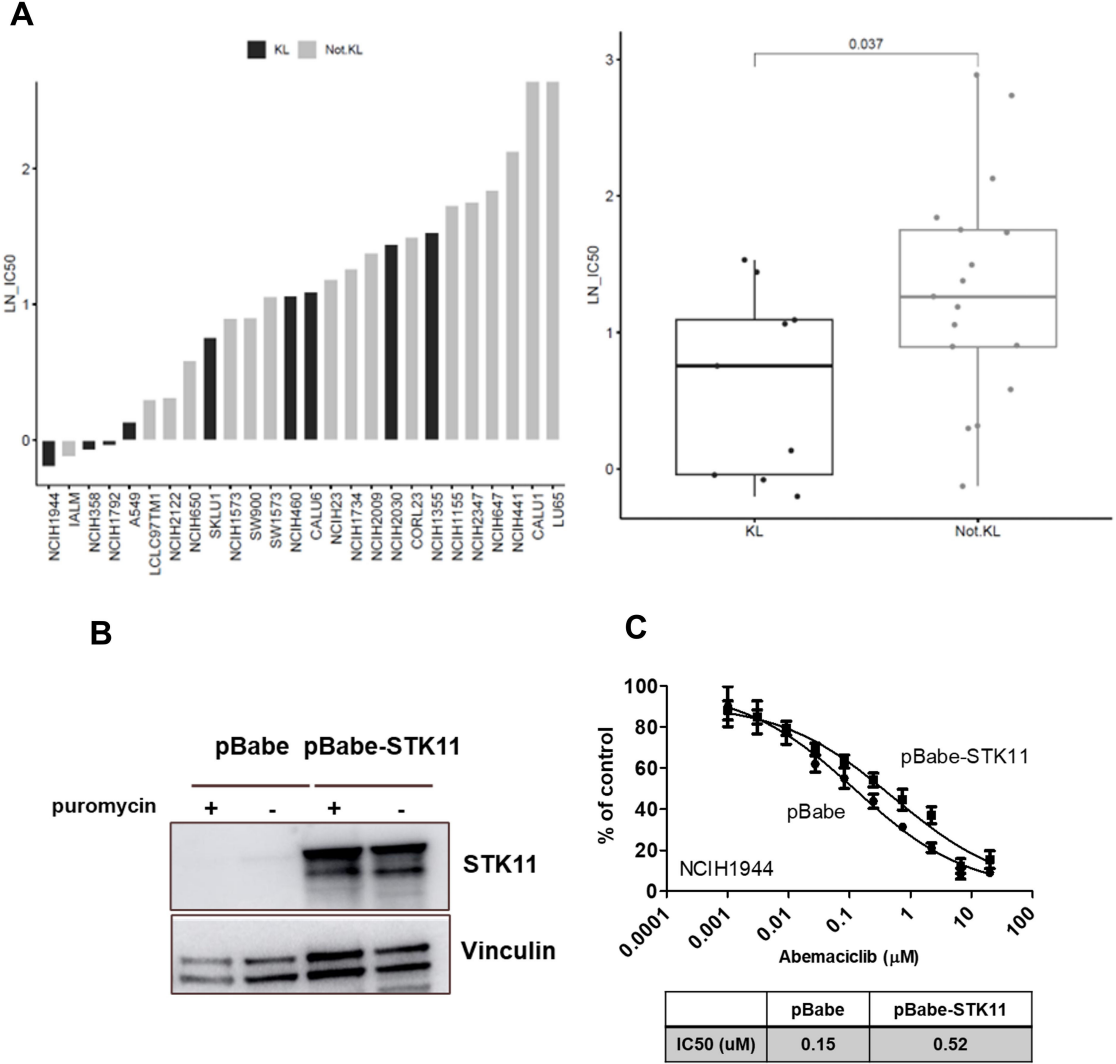
*KRAS*-mutated NSCLC cell line subtypes have differential sensitivity to abemaciclib. IC50 values for the cell lines treated with the CDK4/6 inhibitor abemaciclib. **(A)** Antiproliferative activity of abemaciclib against 24 *KRAS*-mutated NSCLC cell lines. Dot plot shows the log distribution of drug sensitivity to abemaciclib in the KL subtypes and the non-KL subtype (Not-KL = K+KP). Statistically significant differences in the IC50 values of KL compared with non-KL (*P*=.037) as determined by Mann-Whitney U test. Overexpression of *STK11/LKB1* in KL-classified cells (NCI-H1941). Western blots are representative of 2 independent experiments. **(B)** Reduced sensitivity of pBABE-*STK11* NCI-H1941 cells to abemaciclib. **(C)** Cell viability is expressed as % of control (mean ± standard deviation; n=2). CDK, cyclin-dependent kinase; IC_50_, half-maximal inhibitory concentration; *KRAS*, Kirsten rat sarcoma; NSCLC, non–small cell lung cancer; *STK11*, serine/threonine kinase 11.

The abemaciclib activity data from CCSP was used to evaluate differential sensitivity of expression subtypes within the *KRAS*-mutated NSCLC cell lines. Gene expression profiles from cell lines were correlated to centroids identified by Skoulidis et al. (4) for the KL, KP, and K subtypes using the same method used to classify primary tumors. In CCSP, 24 of 43 *KRAS*-mutated cell lines had abemaciclib activity data and were classified: 9 KL, 12 KP, and 3 K subtypes. The 3 K and 12 KP subtype cell lines were combined into the non-KL group to increase statistical power. KL subtype cell lines were more sensitive to abemaciclib treatment than the non-KL subtype cell lines (**Figure 6A**).

The NCI-H1944 cell line with the inactivating *STK11/LKB1* mutation was most sensitive to abemaciclib. To evaluate the relationship between *STK11/LKB1* activity and abemaciclib sensitivity, *STK11/LKB1* was overexpressed in the NCI-H1944 KL cell line, which lacks LKB1 expression (**Figure 6B**). A cell viability assay of the NCI-H1944 KL cell line with *STK11/LKB1* overexpression showed reduced abemaciclib sensitivity, with an approximate 3.5-fold IC50 relative to the parental cell line (**Figure 6C**).

## 4 Discussion

Despite recent advances in *KRAS*-targeted NSCLC therapy, many tumors do not respond to current treatments, including immune checkpoint inhibitors. There are no targeted therapies for *KRAS*-mutant tumors lacking the G12C variant. In particular, *KRAS* plus *STK11/LKB1* co-mutated (KL subgroup) tumors represent an especially poor prognostic subgroup, with lower responses to immune checkpoint and *KRAS* G12C inhibitors (8, 17). Our study results indicate that patients with the expression-derived KL subtype of *KRAS*-mutant NSCLC treated with abemaciclib have significantly improved OS and PFS compared with those treated with erlotinib. This association was further supported by cell line analyses and an *STK11/LKB1* knock-in model. The effect size analyses highlighted meaningful differences across both survival endpoints, with consistent effects and stable variables when subtypes were excluded, supporting the robustness of these findings. These results should be considered preliminary, as they are based on a retrospective analysis of a subset of patients in the JUNIPER trial, where the comparator arm received erlotinib treatment.

We previously reported no association between mutation subgroups and response to abemaciclib in a subset of patients from the JUNIPER trial (17). In contrast, this study identified clinically relevant subgroups and demonstrates a significant association between abemaciclib and clinical outcome in the KL subtype. A possible explanation for this difference is an imperfect association of KL mutation–defined tumors and the associated RNA-defined cluster in earlier reports. The previous analysis examined cancer gene DNA sequencing in a subset of JUNIPER samples after abemaciclib treatment (17). Associated RNA expression data were available for 16 of these genetically *KRAS* plus *STK11/LKB1–*mutated samples. In this sample set, 10 were KL, 1 was KP, and 3 were K subtype tumor samples. Similar to the originally reported data, these results suggest an imperfect association between the genetic (mutation)- and expression (RNA)-derived subtypes. Furthermore, there were differences in clinical parameters associated with abemaciclib treatment between the mutation-defined and expression-defined KL subtypes. The abemaciclib-treated patients with samples characterized as KL by both mutation- and expression-based subtyping (n=10) had an average PFS of 8.42 months, and only 2 patients had an increase in tumor size. In addition, 2 patients experienced PR, and the remaining patients had a best response of SD. In contrast, patients who were classified as having KL subtype tumors by mutation only (n=6) had an average PFS of 1.99 months, an increase in tumor size of ≥14%, and all but one had PD. Another potential explanation for the discordance between the predictive utility of mutation and RNA clustering is that RNA-based clustering performed here was not identical to the original methodology. However, we observed a similar prevalence of each expression-defined cluster with the same relative prognosis as previously reported (4).

Although patients with KL and KP subtype tumors experienced longer PFS with abemaciclib, only those with the KL pattern experienced longer OS after treatment with abemaciclib. The reason for the longer OS observed in patients with the KL subtype is unclear; however, *STK11*/*LKB1* and *CDK4* may play a role in this. The CDK4/6 inhibitor palbociclib was shown to be more potent in cell lines after knockdown of *STK11/LKB1* expression or had decreased sensitivity when *STK11/LKB1* was expressed in *STK11/LKB1*-mutant cell lines (28). Aligned with this observation, we showed that abemaciclib sensitivity was reduced on overexpression of *STK11/LKB1* in a sensitive KL cell line. Furthermore, downregulation of *STK11*/*LKB1* facilitates the G1/S transition, which is regulated by CDK4 (29). These observations are consistent with the increased sensitivity to the CDK4/6 inhibitor abemaciclib seen in tumors with disrupted *STK11*/*LKB1* function associated with KL expression subtype tumors.

In patients with the K subtype, no clinical benefit was observed with abemaciclib compared with that after erlotinib treatment. The K subtype is coupled with low level or no expression of the *NKX2-1*(*TTF1*) transcription factor and higher *CDK*6 expression. *CDK6* amplification or overexpression is one possible resistance mechanism to CDK4/6 inhibitors. Thus, this is consistent with the lack of clinical benefit observed with abemaciclib treatment in this study (30).

Our study had certain limitations that should be acknowledged. First, only a subset of the trial population could be included in the retrospective analysis; this introduced bias. The abemaciclib arm of the TR population had longer OS and PFS than that of the ITT population, while the erlotinib arms were balanced. This bias of the TR population needs to be considered when interpreting the KL expression subtype predictive analysis reported. However, the significance associated with the predictive value relative to the magnitude of the bias suggests the predictive value is relevant. Second, the available clinical data on erlotinib as a comparator in this population are limited and inconsistent. Erlotinib was acceptable in the JUNIPER trial given that it was the only Food and Drug Administration-approved third-line treatment at the time of study initiation and its use was not dependent on the presence of an epidermal growth factor receptor mutation. However, during accrual, in 2016, the Food and Drug Administration label for erlotinib was modified to restrict use to patients with tumors with epidermal growth factor receptor exon 19 deletions or exon 21 (*L858R*) substitution mutations (31). Furthermore, the subsequent availability of immune checkpoint inhibitors changed the second and later line standard of care. Despite the limitations associated with the use of erlotinib as a comparator, docetaxel, a common second-line treatment for *KRAS*-mutant NSCLC, showed similar median OS (7.9 months) and PFS (2.8 months) (32) to what was observed with second- and third-line erlotinib treatment in the JUNIPER trial (OS, 7.8 months; PFS, 1.9 months) (17). Moreover, our investigation focused solely on drug sensitivity within the 2 cell lines. It would be advantageous to incorporate additional cell lines to elucidate the correlation between *STK11/LKB1* activity and sensitivity to abemaciclib.

In conclusion, this retrospective analysis of the phase III JUNIPER trial identified the KL subtype as a candidate predictive biomarker for abemaciclib efficacy in platinum-refractory *KRAS-*mutant lung adenocarcinoma. Although *KRAS* G12C mutations can be targeted, the other *KRAS* mutations, which represent approximately half of *KRAS*-mutated NSCLC, have no approved target-specific therapy. So-called “pan-RAS” and other mutation-specific agents are in development, and if approved, these will be treatment options for subsets of patients with *KRAS*-mutant NSCLC. Our findings can potentially identify a patient subpopulation that may derive a OS benefit from the addition of abemaciclib to best supportive therapy. These findings require independent validation and the development of a robust KL-subtyping companion diagnostic assay before they can be implemented in practice.

## Data Availability

Eli Lilly and Company provides access to all individual data collected during the trial, after anonymization, with the exception of pharmacokinetic, genomic, or genetic data. Access to the data is provided after a proposal has been approved by an independent review committee and after receipt of a signed data sharing agreement. Data and documents (such as study protocol, statistical analysis plan, clinical study report, and blank or annotated case report forms) will be provided in a secure data sharing environment.
