# Supplementary material for: Survival benefit from abemaciclib in non–small cell lung cancer by Kirsten rat sarcoma–mutation gene expression subtype: retrospective analysis from the JUNIPER Trial
Source: Front Oncol. 2025 Mar 31;15:1461530. doi: 10.3389/fonc.2025.1461530 (PMC11994929; doi:10.3389/fonc.2025.1461530)
Supplement: Supplementary file 1 [file DataSheet1.docx]

**SUPPLEMENTARY MATERIAL**

**Supplementary Figure 1.** Kaplan-Meier plots of OS (**A**) and PFS (**B**) in the ITT and TR populations.

**A.**


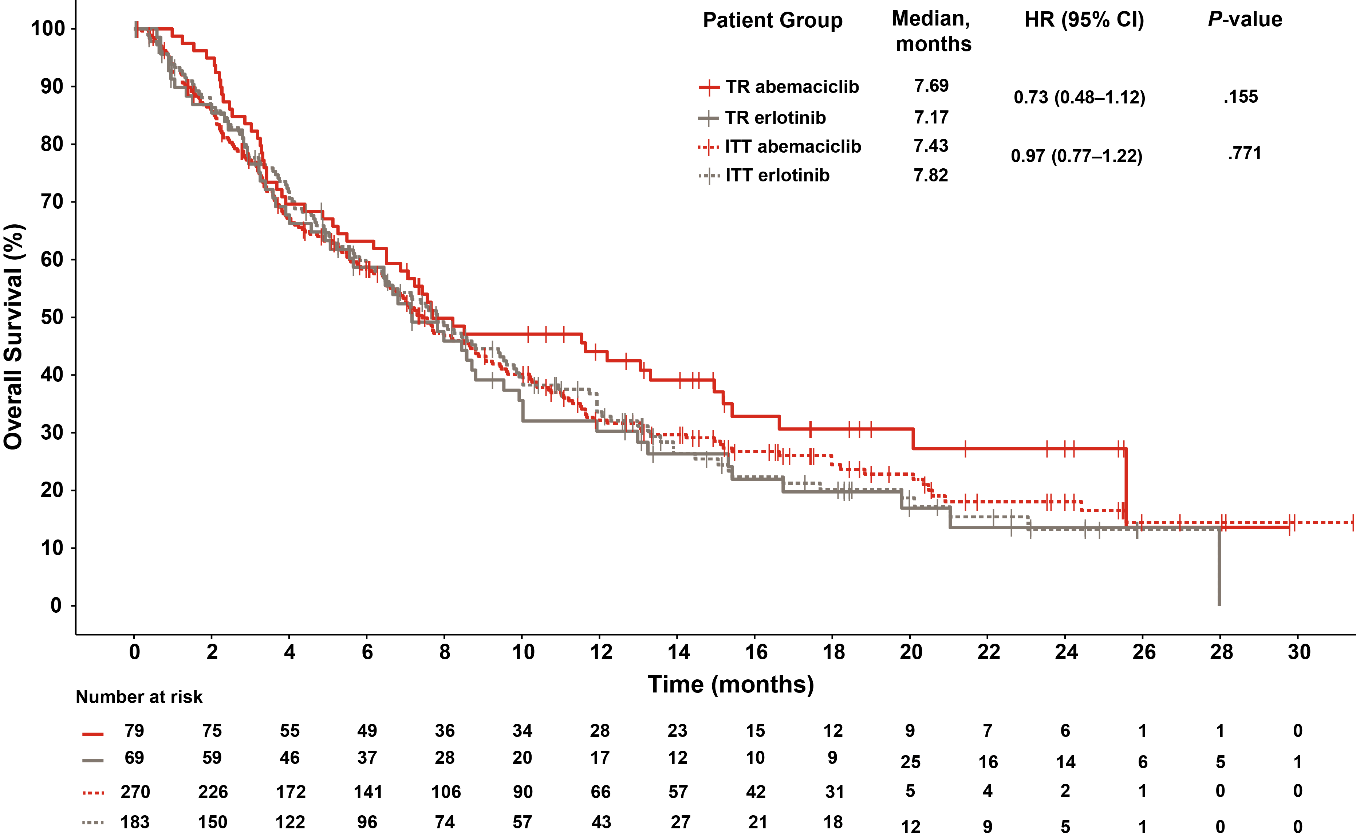


**B.**


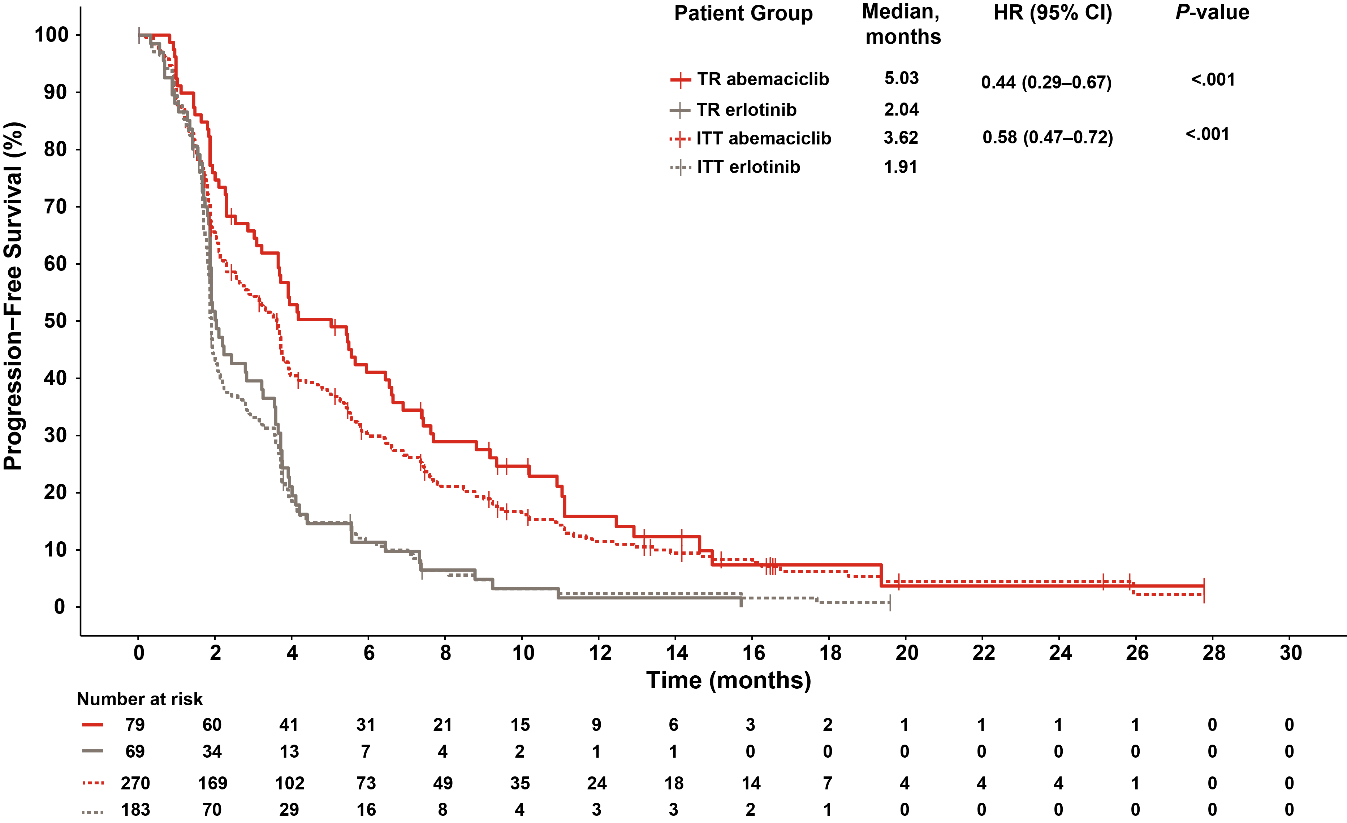


Abbreviations: CI, confidence interval; HR, hazard ratio; ITT, intent-to-treat; OS, overall survival; PFS, progression-free survival; TR, translational research.

**Supplementary Figure 2.** Forest plots for OS treatment effect and univariate subgroup analysis for clinical covariates and stratification factors within the KP subtype (**A**) and K subtype (**B**).


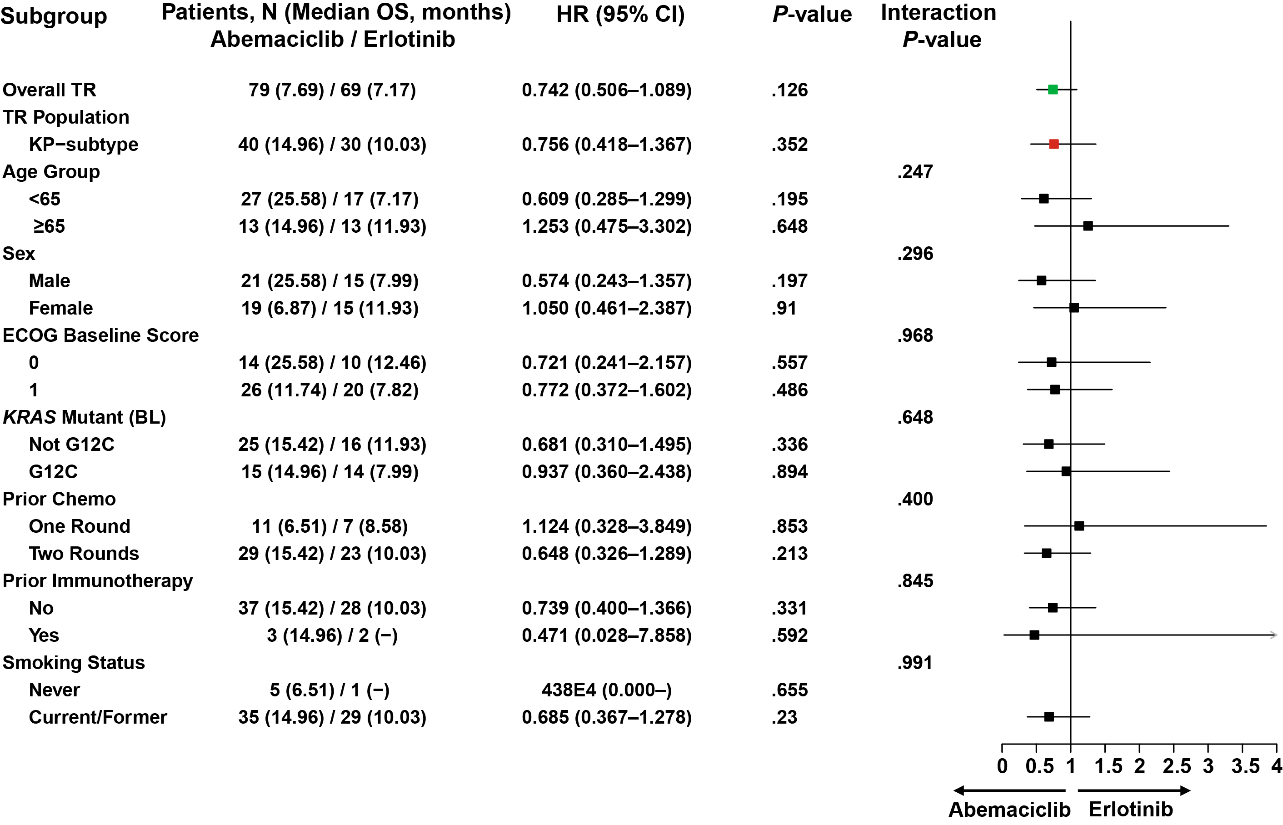


**B**.


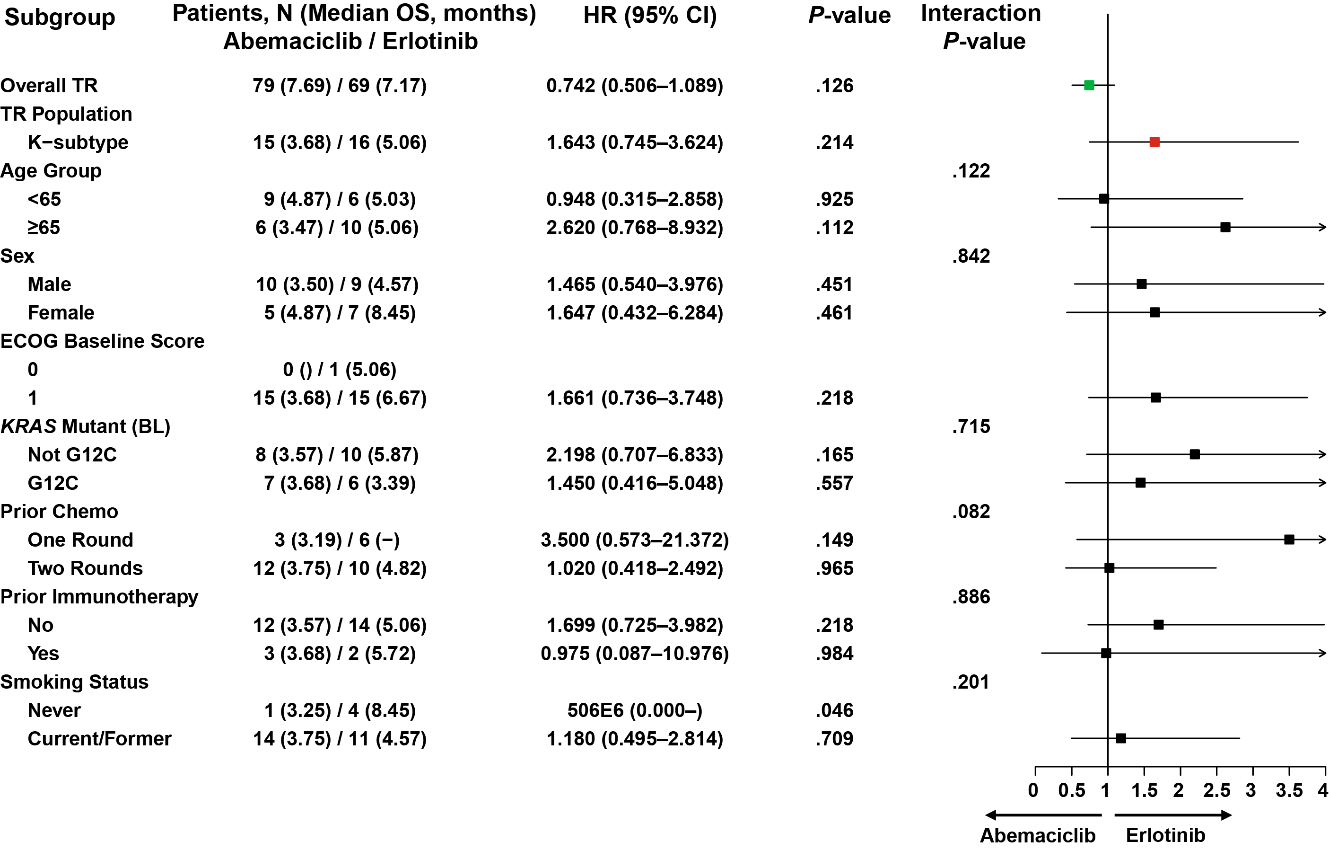


–, Not evaluable due to small sample size; Green: HR for the ITT population; Red: HR for the TR population; Black; subgroups; arrow line, CI out of the x-axis. Univariate analysis was performed to compute the HRs.

Abbreviations: BL, baseline; CI, confidence interval; ECOG, Eastern Cooperative Oncology Group; G12C, mutation in codon 12 of the *KRAS* gene resulting in an amino acid substitution from glycine to cysteine; HR, hazard ratio; *KRAS*, Kirsten rat sarcoma; OS, overall survival; TR, translational population.

**Supplementary Figure 3.** Forest plots showing effect sizes from multivariable analyses for OS and PFS with and without expression subtypes.


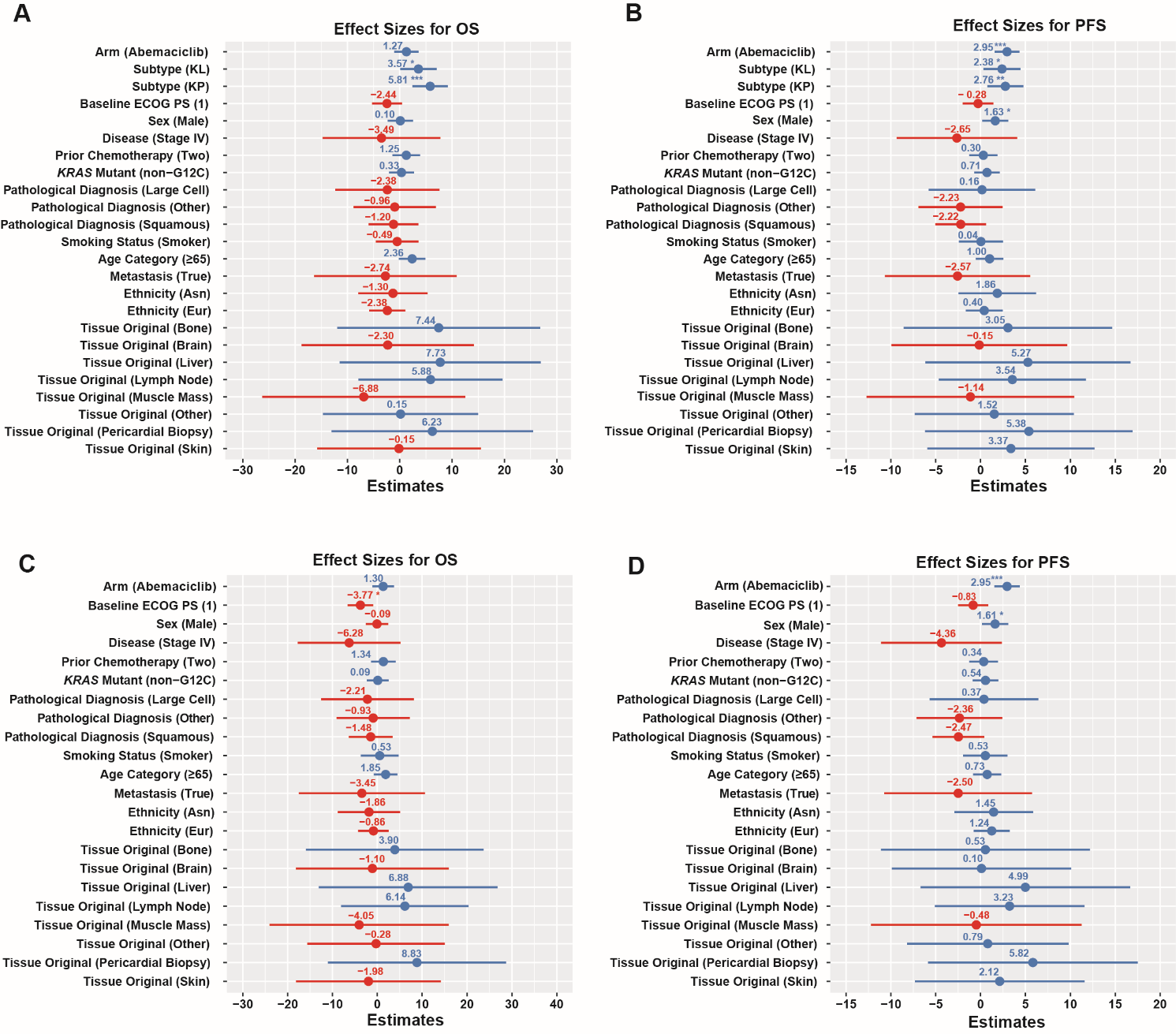


The effect sizes represent the magnitude and direction of the impact that different factors have on the OS or PFS. Panels **A** and **B** show the effect sizes for OS and PFS respectively, including expression subtypes (KP and KL) in the model. Panels **C** and **D** present the corresponding analyses without expression subtypes. Effect sizes are shown as point estimates with confidence intervals. Variables analyzed include tissue of origin, ethnicity, metastasis status, age, smoking status, pathological diagnosis, KRAS mutation status, prior chemotherapy, disease stage, sex, ECOG performance status, and treatment arm (LY2835219). Statistical significance is indicated by asterisks (**P*<.05, **P<.01, *** *P*<.001). Positive values (in blue) indicate increased risk while negative values (in red) indicate decreased risk. Reference categories are not shown in the forest plots.

Abbreviations: ASN, Asian; BL, baseline; ECOG, Eastern Cooperative Oncology Group; G12C, mutation in codon 12 of the KRAS gene resulting in an amino acid substitution from glycine to cysteine; KRAS, Kirsten rat sarcoma; M, male; OS, overall survival; PFS, progression-free survival.

**Supplementary Figure 4.** Multivariate analysis of OS (**A**) and PFS (**B**) in the TR population.


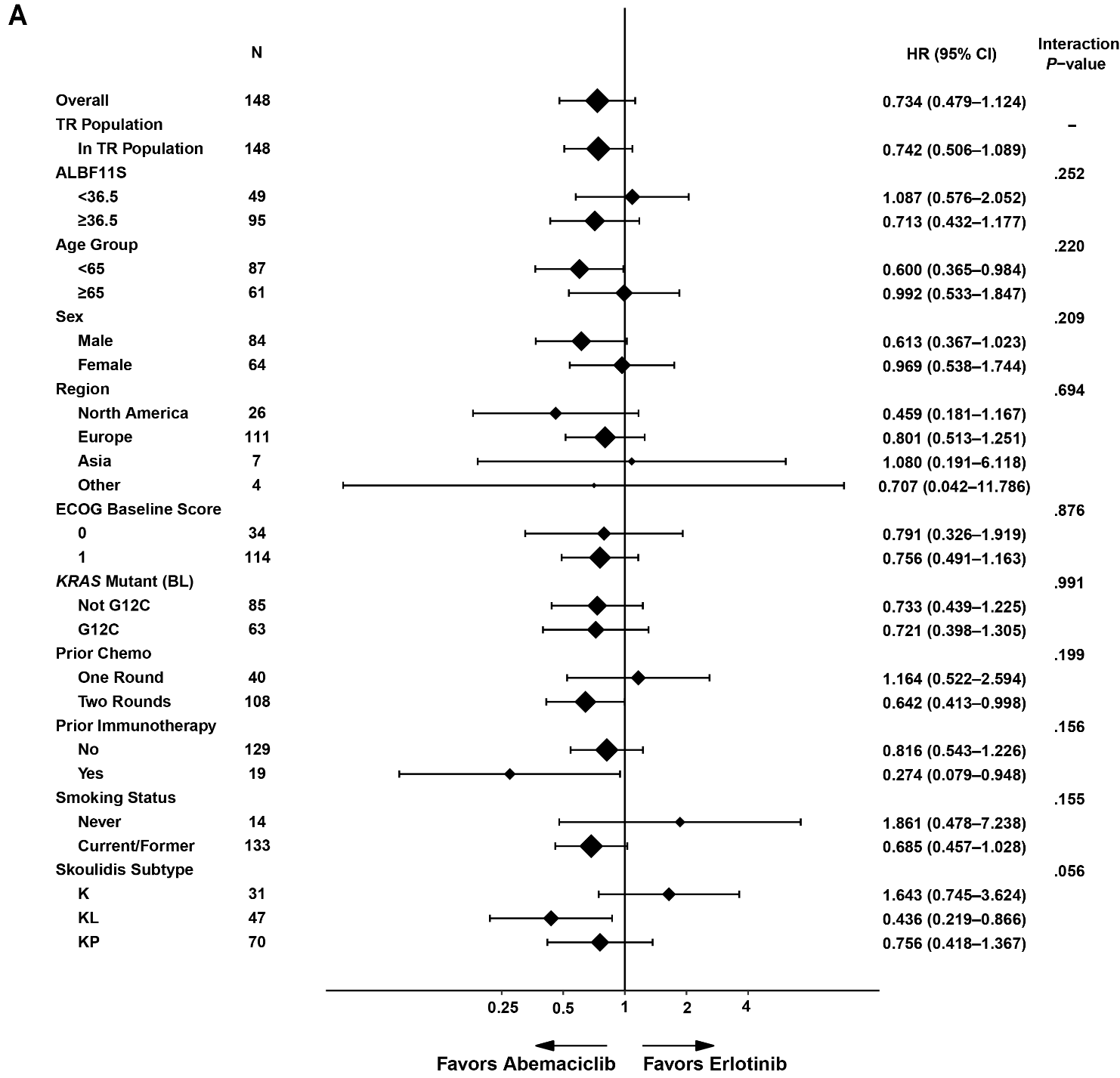


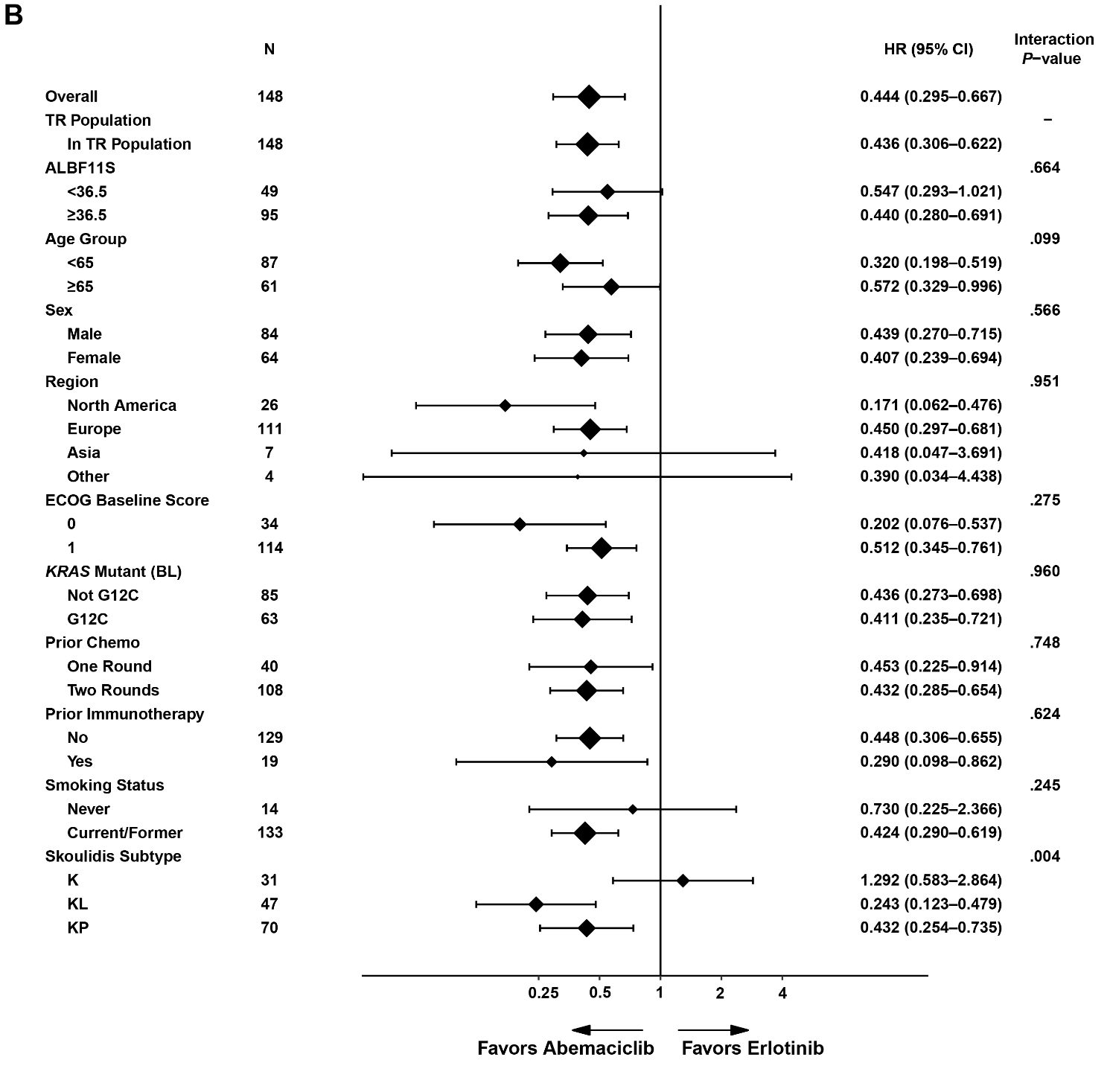


Forest plots showing adjusted HRs with 95% CIs from multivariate Cox proportional hazards models. Each factor was adjusted for all other variables in the model. The analysis includes clinicopathological variables (ALBF11S score, age, sex, geographic region, ECOG performance status, smoking status), molecular characteristics (KRAS mutation status, Skoulidis molecular subtypes), and treatment history (prior chemotherapy, prior immunotherapy ). HRs less than 1 indicate lower risk (better survival), while HRs greater than 1 indicate higher risk (worse survival). N represents the number of patients in each category. Interaction *P*-values assess the statistical significance of each variable in the multivariate model. Treatment effect (abemaciclib vs erlotinib) was evaluated across all subgroups.

Abbreviations: BL, baseline; CI, confidence interval; ECOG, Eastern Cooperative Oncology Group; G12C, mutation in codon 12 of the *KRAS* gene resulting in an amino acid substitution from glycine to cysteine; HR, hazard ratio; *KRAS*, Kirsten rat sarcoma; OS, overall survival; PFS, progression-free survival; TR, translational population.

**Supplementary Table 1.** Multivariable analysis for PFS by expression subtype.

|  | **Subtype KL** | | **Subtype KP** | | **Subtype K** | |
| --- | --- | --- | --- | --- | --- | --- |
|  | **Abemaciclib**  **(N=24)** | **Erlotinib**  **(N=23)** | **Abemaciclib**  **(N=40)** | **Erlotinib**  **(N=30)** | **Abemaciclib**  **(N=15)** | **Erlotinib**  **(N=16)** |
| **Patients censored, n (%)** | 4 (16.66) | 1 (4.34) | 6 (15.00) | 2 (6.66) | 1 (6.66) | 1 (6.25) |
| **Patients with events, n (%)** | 20 (83.33) | 22 (95.65) | 34 (85.00) | 28 (93.33) | 14 (93.33) | 15 (93.75) |
| **Median PFS, months** | 6.64 | 2.10 | 5.52 | 2.24 | 1.94 | 1.87 |
| **HR within expression level (95% CI)** | 0.39 (0.20–0.72) | | 0.21 (0.10­–0.46) | | 0.99 (0.41–2.48) | |
| ***P*-value** | .004 | | .132 | | .468 | |

Sex, ECOG PS, number of prior chemotherapies, and KRAS mutation were used as stratification factors in the model; age, prior immunotherapy, and smoking status were used as covariates in the model.

Abbreviations: CI, confidence interval; ECOG PS, Eastern Cooperative Oncology Group performance status; HR, hazard ratio; N, number of patients in the population; n, number of patients in the category; PFS, progression-free survival.
